# Rapid and highly sensitive detection of pyocyanin biomarker in different *Pseudomonas aeruginosa* infections using gold nanoparticles modified sensor

**DOI:** 10.1371/journal.pone.0216438

**Published:** 2019-07-30

**Authors:** Amal A. Elkhawaga, Marwa M. Khalifa, Omnia El-badawy, Mona A. Hassan, Waleed A. El-Said

**Affiliations:** 1 Department of Medical Microbiology and Immunology, Faculty of Medicine, Assiut University, Assiut, Egypt; 2 Department of Microbiology and Immunology, Faculty of Pharmacy, Assiut University, Assiut, Egypt; 3 Chemistry Department, Faculty of Science, Assiut University, Assiut, Egypt; Institute of Materials Science, GERMANY

## Abstract

Successful antibiotic treatment of infections relies on accurate and rapid identification of the infectious agents. *Pseudomonas aeruginosa* is implicated in a wide range of human infections that mostly become complicated and life threating, especially in immunocompromised and critically ill patients. Conventional microbiological methods take more than three days to obtain accurate results. Pyocyanin is a distinctive electroactive biomarker for *Pseudomonas aeruginosa*. Here, we have prepared polyaniline/gold nanoparticles decorated ITO electrode and tested it to establish a rapid, diagnostic and highly sensitive pyocyanin sensor in a culture of *Pseudomonas aeruginosa* clinical isolates with high selectivity for traces of pyocyanin when measured in the existence of different interferences like vitamin C, uric acid, and glucose. The scanning electron microscopy and cyclic voltammetry techniques were used to characterize the morphology and electrical conductivity of the constructed electrode. The determined linear range for pyocyanin detection was from 238 μM to 1.9 μM with a detection limit of 500 nM. Compared to the screen-printed electrode used before, the constructed electrode showed a 4-fold enhanced performance. Furthermore, PANI/Au NPs/ITO modified electrodes have demonstrated the ability to detect pyocyanin directly in *Pseudomonas aeruginosa* culture without any potential interference with other species.

## 1. Introduction

*Pseudomonas aeruginosa* (*P*. *aeruginosa*) is a prevalent and opportunistic pathogen that is considered one of the most annoying bacteria causing deadly infections in critically ill patients [1–4]. It commonly produces infections in patients with surgical wounds, burn wound or cystic fibrosis. Infections caused by *P*. *aeruginosa* have high morbidity and mortality rates, particularly among immunocompromised patients such as cancer patients and premature infants [5–7]. *P*. *aeruginosa* may acquire multidrug resistance, making its eradication with antibiotics challenging [8]. The increasing resistance of bacteria is partially due to the late diagnosis and the misuse of antibiotics [9]. Hence, the early and fast detection of this serious pathogen is essential for a more targeted antibiotic prescription that will hasten recovery and reduce the emergence of antibiotic resistance [10, 11].

Infections caused by *P*. *aeruginosa* are typically recognized in laboratories by using selective culturing techniques, which needs at least 24 h to obtain the results. Furthermore, the polymerase chain reaction (PCR) is utilized for the diagnosis of *P*. *aeruginosa*. However, PCR needs expensive chemicals, require complicated sample preparations, and is a time-consuming technique [12]. Therefore, developing a sensitive, specific, and rapid identification methods for pathogen detection in cost and time competent manners have found broad attention in the last few years [13]. Pyocyanin is one of the virulence factors exclusively secreted by *P*. *aeruginosa*. It is a unique, quorum-sensing molecule that is linked to biofilm formation, induces inflammation, and causes apoptosis of neutrophils [14–16].

Until lately, pyocyanin was measured by chromatography or spectrophotometric techniques that are time-consuming and needs purification from bacterial cultures [15]. Electrochemical sensors are recommended efficient tools for the identification of different important biological, chemical, and environmental targets [13]. They are easy to use with low detection limits, excellent sensitivity, good stability, together with cost and time effectiveness [17, 18]. The redox-active nature of pyocyanin molecules permits its rapid detection by electrochemical sensors in two minutes [19]. It is still a challenge to find new and more sensitive methods to provide timely and accurate information about *P*. *aeruginosa* that can aid prompt treatment decisions when bacteria are stil responding to antibiotics treatment.

Gold (Au) nanostructures are currently used to modify electrodes of biosensors because of its excellent optical and electrical properties and affinity to bind with biomolecules [20–27]. Moreover, the Au nanostructures decorated indium tin oxide (ITO) substrate was successfully used to detect several multidrug-resistant bacteria [28].

Conducting polymers [29] have gained much interest in recent researches because of their excellent conductivity, stability and ease of preparation. In general, the electronic and electrochemical properties of [30–33] conducting polymers made them have many applications in photovoltaic cells, organic light emitting diode, and biosensors. Polyaniline (PANI) has received much attention in the research work. This is mainly because PANI and its derivatives or composites with other materials are easy to synthesize chemically or electrochemically [34].

Therefore, conducting polymers/metal or metal oxides hybrid materials possess the unique combination of the conducting polymers properties (biocompatibility, direct electrochemical synthesis) and the unique features of nanomaterials including large active surface area, flexibility for the immobilization of biomolecules and the quantum effect that could enhance the rate of the electron transfer of the developed sensor [35]. Recently, we have manufactured poly(4–aminothiophenol) nanostructures coated Au nanodots ITO electrode for highly sensitive and selective electrochemical detection of a mixture of adenine and guanine DNA bases [24].

Hybrid organic and inorganic nanocomposites not only possess the sum of their individual components, but also the role of the inner interfaces could be predominant; thus hybrid organic/inorganic nanocomposites have been broadly used in a variety of applications including biosensors [22, 36–40] and sensors [41].

The present work aims to assess the efficacy of using PANI/Au nanostructures modified ITO sensor for early detection and quantification of pyocyanin in *P*. *aeruginosa* cultures of clinical isolates based on cyclic voltammetry (CV) technique.

## 2. Methods

### 2.1. Materials

Pyocyanin (P0046-5MG), ITO substrates and gold (III) chloride hydrate (HAuCl_4_) were purchased from Sigma Aldrich. Deionized water (DIW) with a resistivity of 18.2 MΩ.cm was used for all preparations. Phosphate buffer saline (PBS) (0.01 mol/L, pH 7.4) was prepared by dissolving PBS powder in 1 L of DIW. Luria-Bertani (LB) broth (Oxoid, UK) was utilized in this study.

### 2.2. Instruments

The Autolab potentiostat instrument (Netherlands) connected with the three-electrode cell was used for all electrochemical measurements; Metrohm Model 663VA stand was controlled using Nova software at room temperature. The three-electrode system comprised of a counter electrode of a platinum wire, a reference electrode of Ag/AgCl and a working electrode of PANI/Au modified ITO electrode.

### 2.3. Pseudomonas aeruginosa clinical isolates

*P*. *aeruginosa* cultures were made from clinical isolates obtained from the Department of Medical Microbiology, and Immunology that were isolated from clinical cases of *P*. *aeruginosa* infections admitted to Assiut University hospital as pneumonia, corneal ulcers, urinary tract infections, and wound infections. The clinical isolates of *P*. *aeruginosa* were proved by using the VITEK 2 automated microbiology system. The study protocol was approved by the local Ethical Committee of the Faculty of Medicine, Assiut University, (IRB no: 17300293) and informed written consent was taken from all the study participants.

### 2.4. Methods

#### 2.4.1. Synthesis of gold nanostructures decorated ITO electrode

The ITO electrode with a size of 2.5 cm X 1.25 cm was first cleaned by sonication in 1X Triton, DIW and then in ethyl alcohol for 15 min each. The substrates were immersed in a basic piranha solution, which consists of a mixture of H_2_O_2_:NH_3_: H_2_O (1:1:5 v/v) for 30 minutes at 80°C. Finally, the electrodes were rinsed with DIW and ethanol and dried under nitrogen gas. The Au NPs modified ITO electrodes were prepared by following our previously described method [21]. An aqueous solution of 0.001 M of gold chloride was added into the electrochemical cell and we have issued the deposition process of Au NPs on the ITO substrates by using CV technique within potential window from 1.5 V to -1 V for 5 cycles at scan rate of 50 mV/sec against Ag/AgCl reference electrode. The morphology of the modified electrode was investigated by SEM (JOEL-JSM-5400LV).

#### 2.4.2. Preparation of gold modified ITO electrode

Polyaniline hydrochloride (PANI) salt was prepared according to the previously published work [42]. Typically, 0.5 g of aniline hydrochloride was dissolved in 20 mL of DIW and stirred in an ice bath for 1h (first solution). In another conical flask, a solution of 0.5 g of ammonium persulphate in 20 mL DIW was stirred in an ice bath for 1h and then added to the first solution with stirring for 4 hours. The dark green precipitates were filtrated and dried in an oven at 80°C [43]. To fabricate a layer of PANI on the surface of Au nanostructures decorated ITO electrode, the modified electrode was immersed in a solution of PANI in NMP (0.001 gm/mL) for 8 hrs and then rinsed with DIW to remove the PANI from the non-conductive side and dried under N_2_ gas [42, 44].

#### 2.4.3. Electrochemical measurements of pyocyanin

The electrochemical cell was charged with different concentrations of pyocyanin within a range from 1.9 to 238 μM in PBS (10 mmol/L, pH 7.4) as an electrolyte. Then, working electrode together with the reference and counter electrodes were immersed in the pyocyanin for the electrochemical measurements.

#### 2.4.4. The selectivity of the developed pyocyanin sensor

The electrochemical responses of several mixtures of pyocyanin with different interferences including glucose, vitamin C, and urea as common interferences present in clinical samples has been investigated to validate the selectivity of the developed biosensor towards the pyocyanin biomarker.

#### 2.4.5. Electrochemical detection of pyocyanin in Pseudomonas aeruginosa cultures

Under complete sterile conditions, a colony (or more) of *P*. *aeruginosa* were added in 10 ml of LB broth in 14 ml tube and placed on a shaker (200 rpm) at 37°C overnight. 1ml of this suspension was removed and added to 9 ml fresh LB broth in 14 ml tube and placed again in a shaker at 37°C for one day. Three samples were collected at different time-points (after 2, 10 and 24 hours) during the 24 hours. The concentration of pyocyanin was estimated using the PANI/Au NPs modified ITO electrode. The OD at 600 nm (OD600) was measured to quantify the density of the bacteria and confirm the increasing bacterial number in each culture sample.

#### 2.4.6. Electrochemical testing of bacterial cultures

Each strain of *S*. *epidermidis*, *S*. *aureus*, *S*. *pneumonia*, *P*. *aeruginosa*, *and K*. *pneumoniae* was cultivated in LB broth at 37°C overnight. The cyclic voltammetry of each strain culture was measured using the PANI/Au NPs modified ITO electrode.

#### 2.4.7. Electrochemical detection of P. aeruginosa isolated from clinical samples

Different *P*. *aeruginosa* clinical isolates were collected for electrochemical testing. Samples were cultivated in LB broth and incubated at 37°C overnight. The CV of each sample was measured using the PANI/Au NPs modified ITO electrode.

## 3. Results and discussion

### 3.1 Synthesis and morphological features of the Au nanostructures/ITO and PANI/Au NPs/ITO electrodes

**Fig 1:**
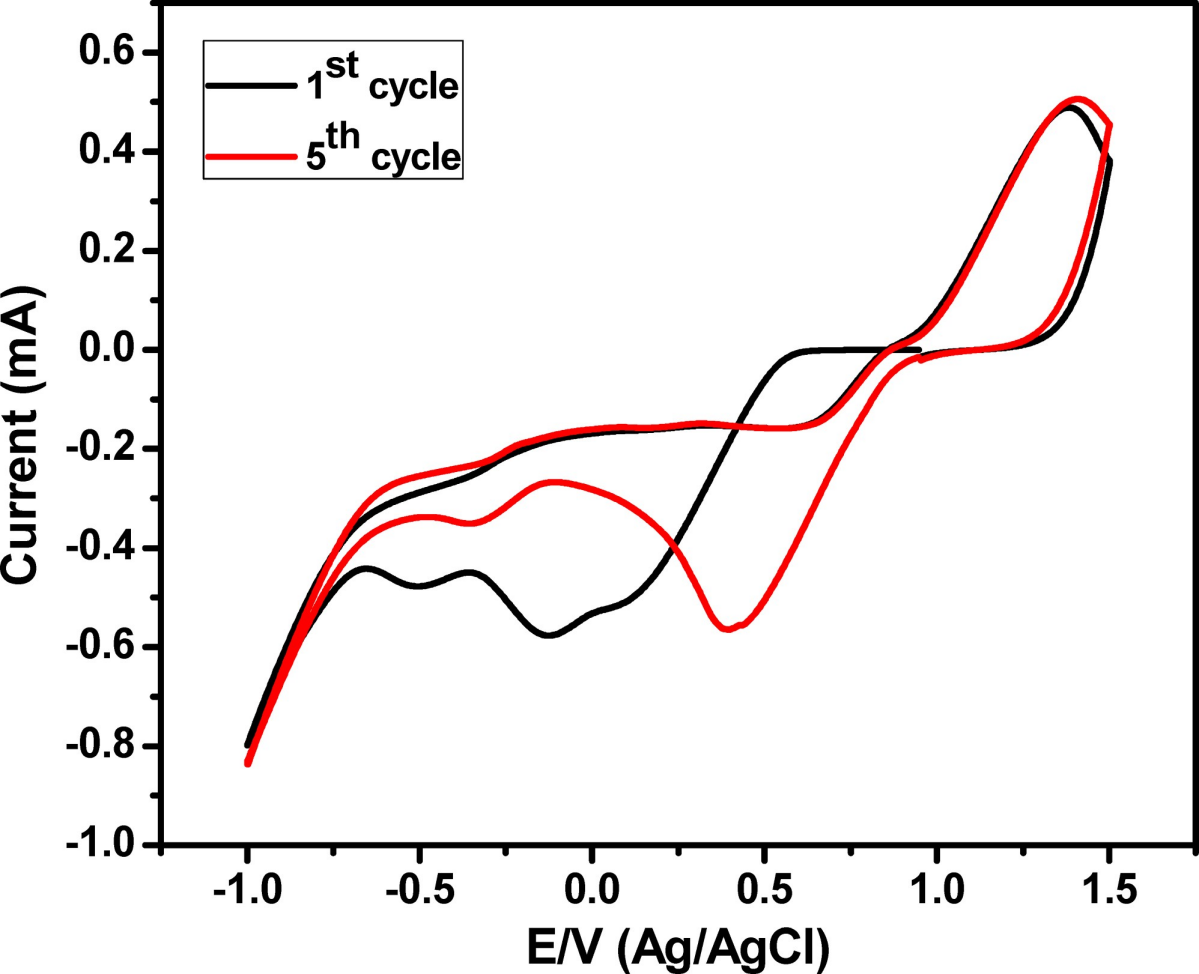
Electrochemical deposition of Au NPs onto ITO substrate based on CV technique within a potential window from 1.5 V to -0.1 V, scan rate was 50 mV/sec.

In the present work, the preparation of Au NPs decorated ITO electrode was performed *via* electrochemical deposition of Au nanostructures onto the ITO substrate based on CV technique. The electrochemical deposition process was performed within a potential window from 1.5 V to -1.0 V for 5 cycles to allow a complete reduction of Au^3+^ ions into Au^0^ [20]. **Fig 1** demonstrated a reduction peak at -0.5, -0.13 and 0.1 V, anodic peak at -0.1 and 0.84 V during the first cycle of Au nanoparticles deposition, which is shifted to -0.35 & 0.41 V; in addition to oxidation peaks at 0.061 & 0.84 V during the deposition process in the remaining 4 cycles. The shifting in the reduction peaks is related to the reduction process of Au^3+^ to form metallic Au nanostructures on the ITO electrode surface [20].

**Fig 2:**
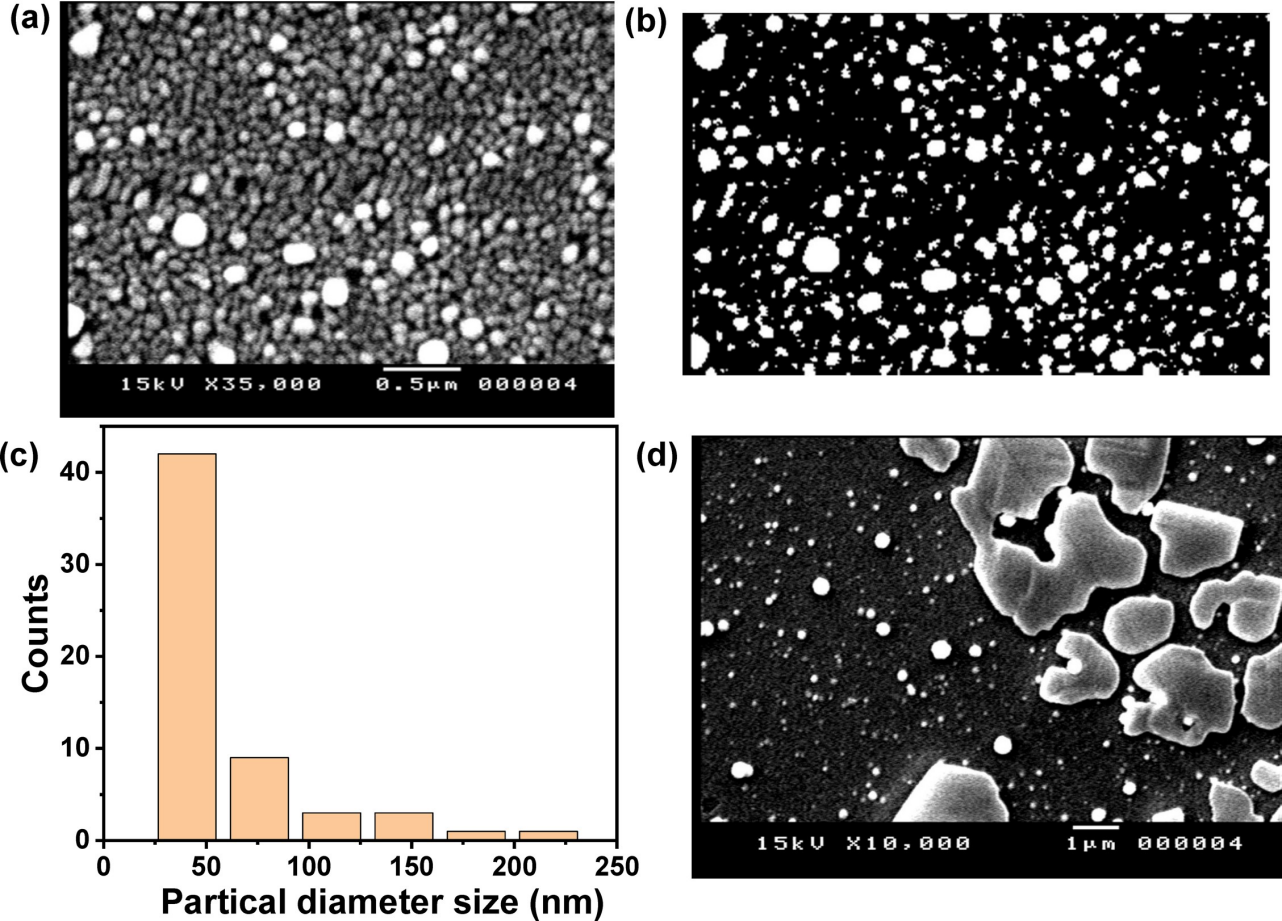
(a) SEM image of Au NPs modified ITO electrode prepared after 5 cycles, (b) ImageJ (IJ 152) analysis of the SEM image of the Au NPs modified ITO electrode prepared after 5 cycles, (c) the distribution of particle diameter size for the analyzed 59 particles and (d) SEM image of PANI/ Au NPs/ITO electrode.

The morphology of the Au nanostructured coated ITO electrode was explored by SEM as it could have a significant effect on the electrochemical detection sensitivity. The SEM image of the Au nanostructured decorated ITO electrode (**Fig 2A**) illustrated the formation of Au NPs with circle morphology and the diameter size was ranging from 76.9 nm to about 192.3 nm and thus we have obtained polydispersed particles. Furthermore the SEM image showed the distribution of Au NPs which indicated the excellent coverage of Au nanostructures over the ITO electrode surface. The average particle size was analyzed by using ImageJ (version IJ152) program (**Fig 2B**), which analyzed the data of 59 particles and the results demonstrated that the mean particle size was found to be about 75.26 nm in diameter with a standard deviation of about 42.737 nm. **Fig 2C** showed the distribution of particle diameter size for the analyzed 59 particles, which indicated that most of the analyzed particles have small diameter with few particles with large diameter.

### 3.2. The electrochemical response of pyocyanin biomarker by using cyclic voltammetry

**Fig 3:**
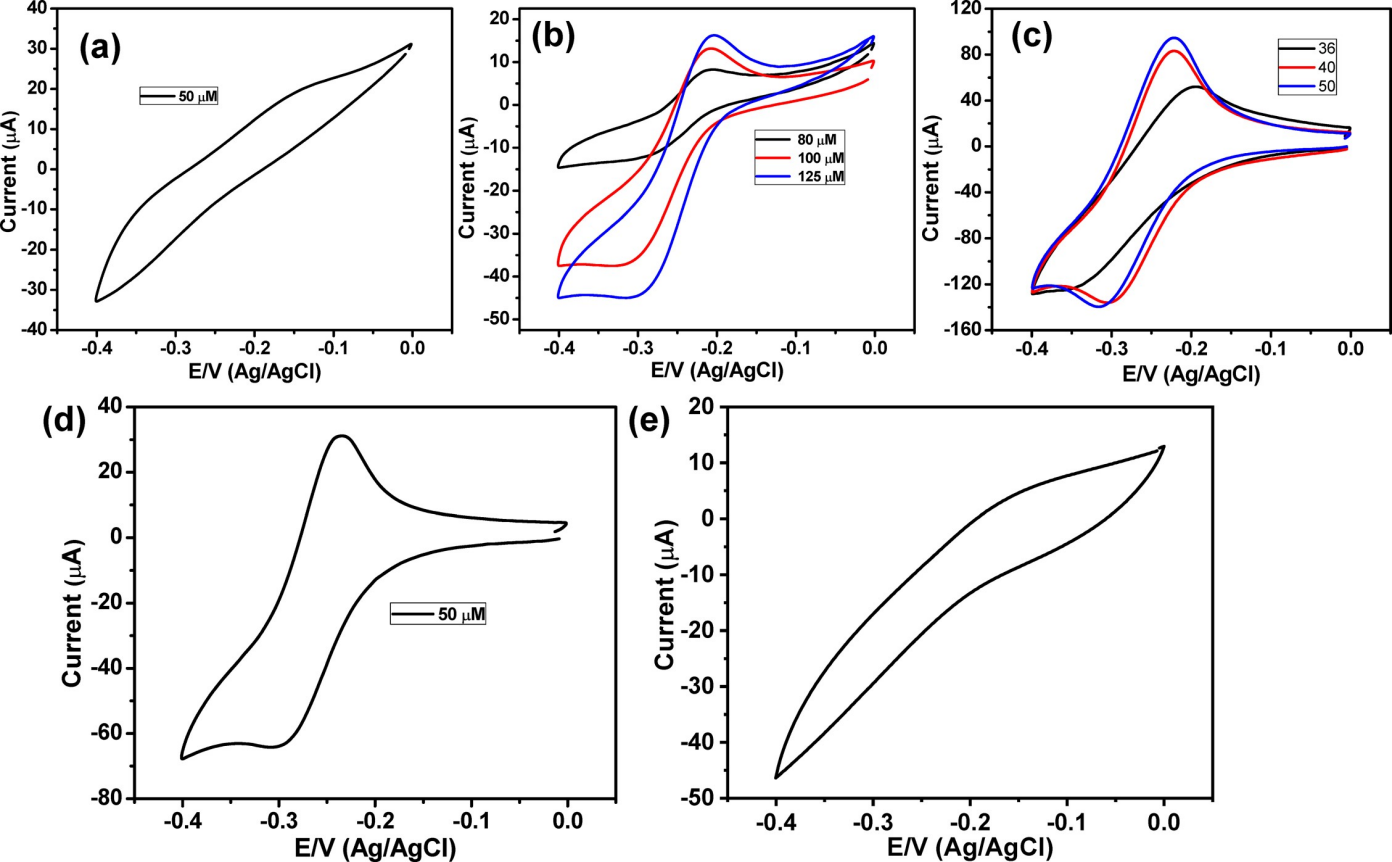
Cyclic voltammetry behavior of (a) 50 μM pyocyanin in PBS buffer at bare ITO, (b) three different concentrations of pyocyanin in PBS buffer at bare ITO, (c) three different concentrations opyocyanin in PBS buffer at Au NPs modified ITO, (d) 50 μM pyocyanin in PBS buffer at PANI/Au NPs modified ITO and (e) 50 μM pyocyanin in PBS buffer at PANI/ modified ITO. The scan rate was 50 mV/sec.

**Fig 3A** represented the CV behavior of 50 μM of pyocyanin in PBS at the bare ITO electrode, which demonstrated very weak redox peaks. So, the CV responses of higher concentrations were investigated (**Fig 3B**) that exhibited an oxidation peak at -0.203 V and a reduction peak at -0.305 V. Thus, the bare ITO electrode is unsuitable for detecting low concentrations of pyocyanin. To develop an electrode that could sense the pyocyanin; the ITO electrode was modified with Au nanostructures and was used to study the electrochemical behavior of pyocyanin based on CV method. **Fig 3C** presented the cyclic voltammograms of three different concentrations of pyocyanin at Au NPs modified ITO electrode, which illustrated a quasirevisable response with one oxidation peak at -0.21 V and a reduction peak at about -0.3 V. These results showed the ability of the Au NPs coated ITO electrode fo pyocyanin detection; this capability is attributed to the signal strengthening of Au nanostructures that improved the rate of the electron transfer [21, 45]. However, the Au NPs modified ITO electrode didn’t show any response to pyocyanin solution with concentrations lower than 36 μM. To enhance the sensitivity of the developed electrode, we have modified the Au nanostructured/ITO electrode with a layer of PANI and then used it to detect the pyocyanin marker. **Fig 2D** showed the SEM micrograph of the PANI/Au NPs/ITO, which showed the formation of a thin layer of PANI with a large diameter in addition to the presence of uncovered Au NPs. This results confirmed the fabrication of PANI layer over the Au NPs and the formation of PANI/Au NPs core/shell. The cyclic voltammogram of 50 μM pyocyanin at PANI/Au NPs/ITO electrode was exhibited in **Fig 3D** that showed an increase in the anodic peak at -0.23 V and cathodic peak at -0.3 V. Furthermore, it is interesting to note that the redox current peak is higher than that in either case of using ITO or Au NPs/ITO electrodes. In addition, the revisability of the redox behavior was increased with electrode modification. This indicates that PANI/Au NPs/ ITO electrode is more sensitive to pyocyanin than either bare ITO electrode or Au NPs/ITO electrode.

To study the role of the PANI layer, we have fabricated PANI/ITO electrode based on electrochemical polymerization method and then using this electrode for monitoring the pyocyanin biomarker. **Fig 3E** represented the CV response of 50 μM pyocyanin at PANI/ITO electrode, which showed a broad background with an anodic current peak at about -0.14 V but no cathodic peak could be observed. These results indicated that although the PANI/ITO electrode could show the anodic peak but the rate of electron transfer was not enough to show the cathodic peakand hene we need to develop more senstive electrode.

**Fig 4:**
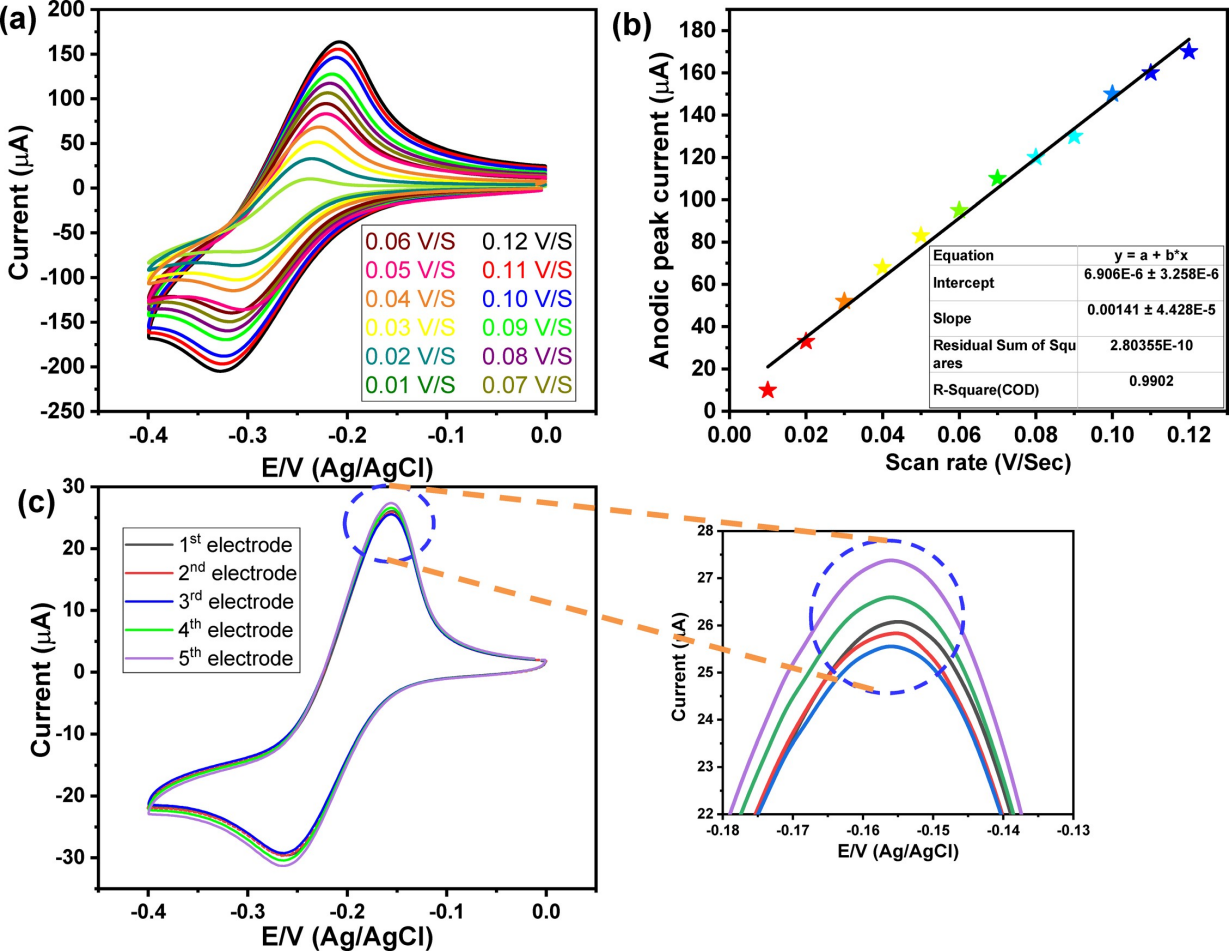
a) CV of pyocyanin 50 μM at different scan rate from 0.01 V/s to 0.12 V/s, b) scan rate versus the oxidation peak current of pyocyanin, and c) CV response of 30 μM of pyocyanin at five different PANI/Au NPs/ITO electrodes at scan rate of 50 mV/sec.

**Fig 4A** showed the scan rate effects within a range from 10 mV/s to 120 mV/s on the maximum current peak of pyocyanin, which illustrated an increase in the redox peaks with the rise of the scan rate. **Fig 4B** illustrated the relationship between the value of the scan rate versus the maximum peak current of pyocyanin at Au NPs/ITO electrode, which demonstrated a linear relationship over a wide range of scan rate from 0.01 V/s to 0.12 V/s.

### 3.3. The reproducibility and the sensitivity of the developed sensor towards pyocyanin marker

Before using the modified electrode for detecting different concentrations of pyocyanin, we have studied the reproducibility of our data based on fabricated five electrodes under the same conditions and used them to measure the same pyocyanin concentration. **Fig 4C** showed the cyclic voltammograms of 30 μM of pyocyanin at five PANI/Au NPs/ITO electrodes, which showed almost the same anodic current peaks (25.58, 25.84, 26.01, 26.633 and 27.41 μA). The maximum variation in the current peak was about ±0.5 μA between the different five electrodes. These results indicated the high reproducibility of these electrodes.

**Fig 5:**
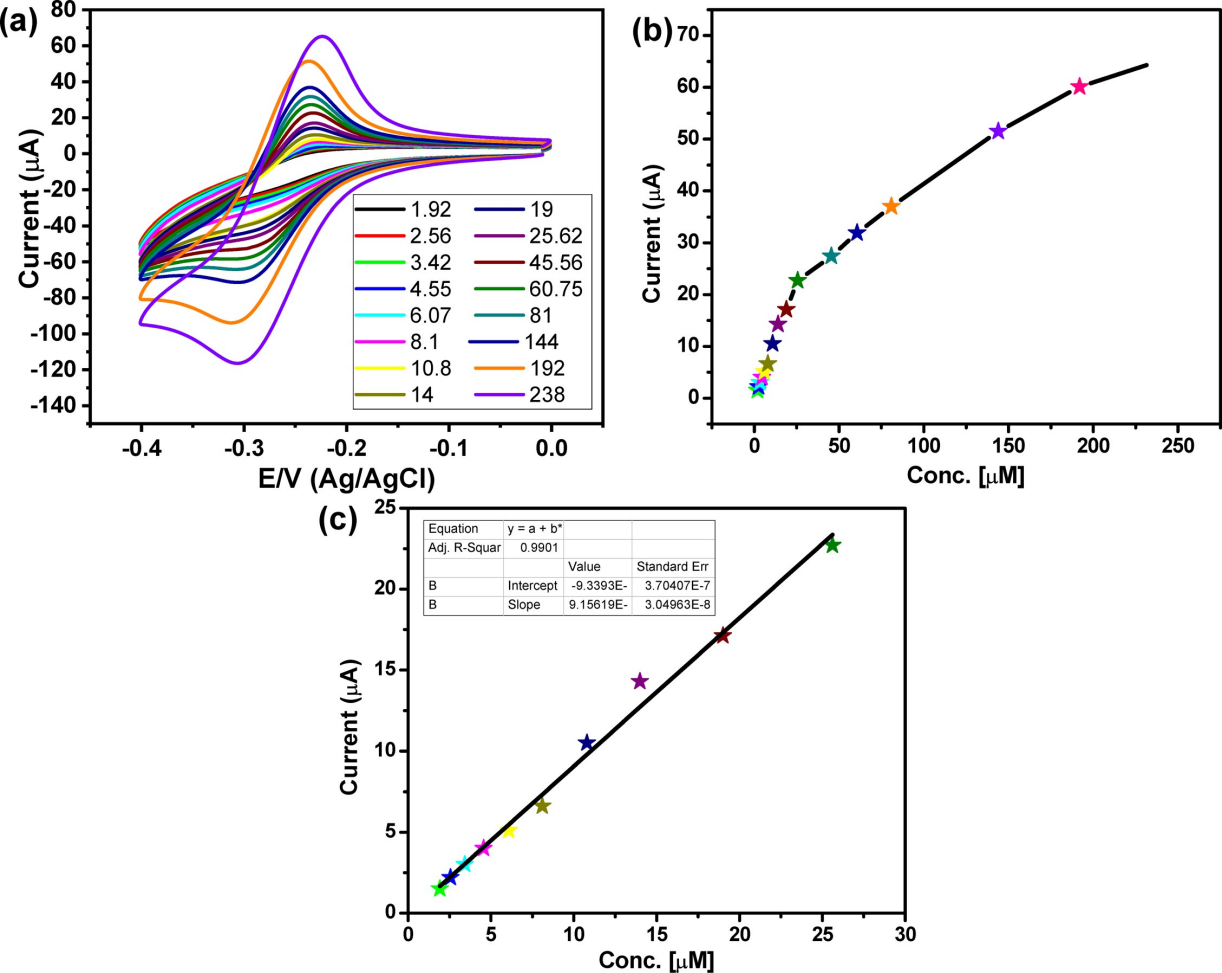
(a) Cyclic voltammograms of different concentrations of pyocyanin from 238 𝜇M to 1.9 𝜇M at scan rate 50 mV/sec, (b) the relationship between the anodic current peaks and the pyocyanin concentration, and (c) linear relation between current peak and pyocyanin concentrations from 25.62 𝜇M to 1.9 𝜇M.

To assess the sensitivity of the PANI/Au NPs decorated ITO electrode towards pyocyanin, the CVs responses of a wide range of pyocyanin concentrations (from 1.9 μM to 238 μM) in PBS were investigated at PANI/Au NPs modified ITO electrode. **Fig 5A** showed the CVs performance of the pyocyanin different concentrations at PANI/Au NPs modified ITO electrode, which showed an increase in the redox current peaks with the increase in the pyocyanin concentration. **Fig 5B and 5C** represented the oxidation current peak vs the pyocyanin concentration at PANI/Au NPs decorated ITO electrode, which illustrated a linear response between the anodic current peaks and the concentration of pyocyanin. The LOD of the PANI/Au nanostructured modified ITO electrode was calculated by following the equation LOD = 3.3*(STEYX/Slope of calibration curve), and it was 500 nM. This result is better than the results obtained by Alatraktchi *et al*., who detected pyocyanin by a disposable screen-printed electrode [46]. The results in **Fig 5** showed the high sensitivity for detection of pyocyanin using the Au modified ITO, which was attributed to the use of PANI/Au nanostructured decorated ITO as a working electrode that achieved the precise, rapid and sensitive measurement of pyocyanin with a low cost. **Table 1** showed the LOD of our modified electrode in comparison with the LOD of some previously reported electrodes for the electrochemical pyocyanin determination. The results in the table showed that the present electrode possessed a lower LOD in contrast with most of the previously reported electrodes [46–52], although they have used more sensitive electrochemical techniques including the square wave voltammetry (SWV) and differential plus voltammetry (DPV).

**Table 1:** Comparison between the sensitivity of our sensor with the previous work.

Sensor	Electrochemical technique	LOD	References
Screen-printed electrode (gold working electrode)	CV	2 μM	**(44)**
Screen-printed electrode (gold working electrode)	Amperometry	0.125 μM	**(45)**
Screen printed sensing glove (carbon ink)	SWV	0.003 μM	**(46)**
Three electrode configurations consisting of a Carbon Fibre tow laminate working electrode	SWV	0.030 μM	**(47)**
Paper-based sensor (carbon electrode)	SWV	0.095 μM	**(48)**
Boron-doped diamond (BDD) thin-film electrode	DPV	0.05 μM	**(49)**
-T-Macro	SWV	0.51 μM	**(50)**
-1.54T-CUA	SWV	1.0 μM	**(50)**
-CS/GNP 1.54T-CUA	SWV	1.6 μM	**(50)**
PANI/Au NPs/ITO	CV	0.5 μM	**The present work**

### 3.4. The selectivity of the sensor towards pyocyanin in the presence of different interferences

**Fig 6:**
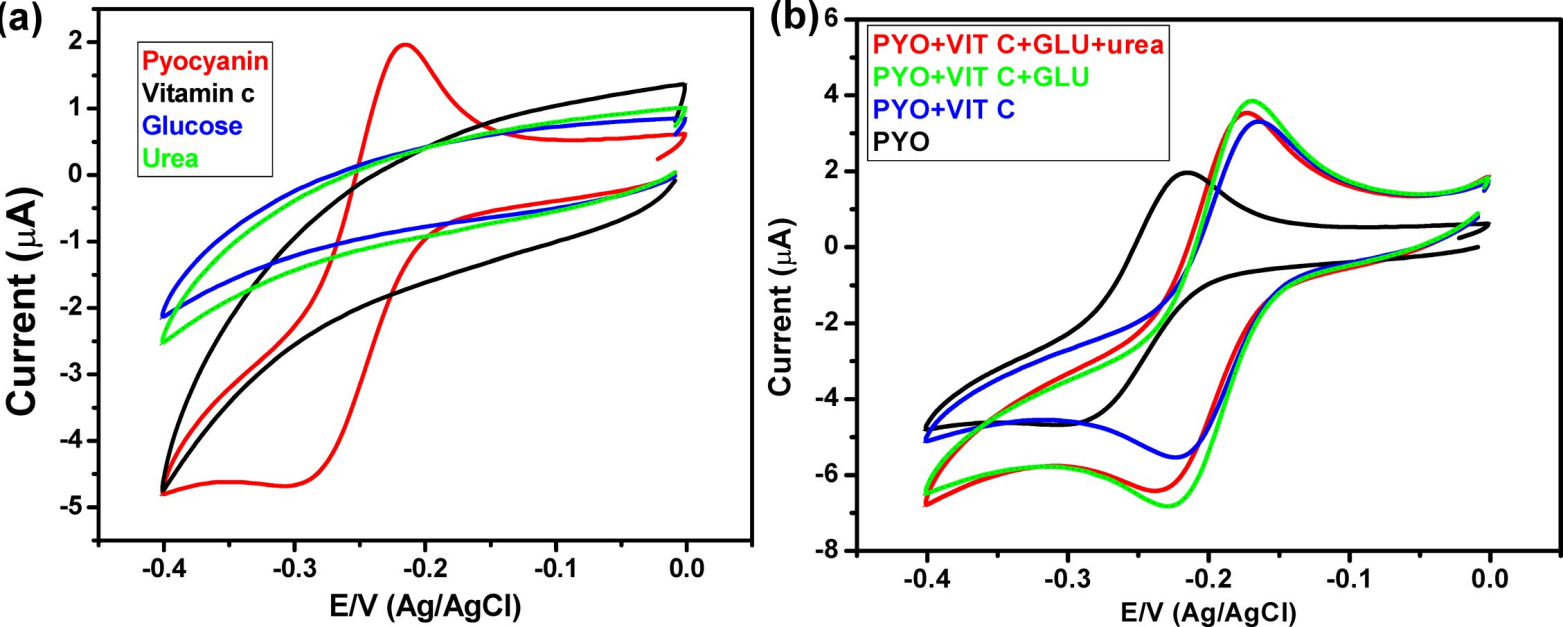
(a) CV of pyocyanin, vitamin C, glucose and urea, and (b) CV of pyocyanin and various mixtures of pyocyanin, vitamin C, glucose and urea.

One of the concerns raised about the utility of the PANI/Au nanostructured coated ITO electrode for investigating the existence of *P*. *aeruginosa* in human samples is that there may be other molecules, which may interfere with electrode performance. It is crucial to achieve high selective and sensitive sensing efficiency against pyocyanin in the presence of different interferences in clinical samples. This study focused on the selective detection of pyocyanin in the existence of vitamin C, glucose, and urea, which may be present in clinical samples. **Fig 6** displayed the CV of pyocyanin and interferences; there are no peaks of interferences in the potential window of pyocyanin. The obtained results revealed that the selective detection of pyocyanin is applicable, with high sensitivity. The results reported in **Fig 6** showed a clear electrochemical fingerprint of pyocyanin, which was observed when pyocyanin was measured among other redox-active compounds such as vitamin C, urea, and glucose.

### 3.5. Electrochemical pyocyanin detection in Pseudomonas aeruginosa culture

**Fig 7:**
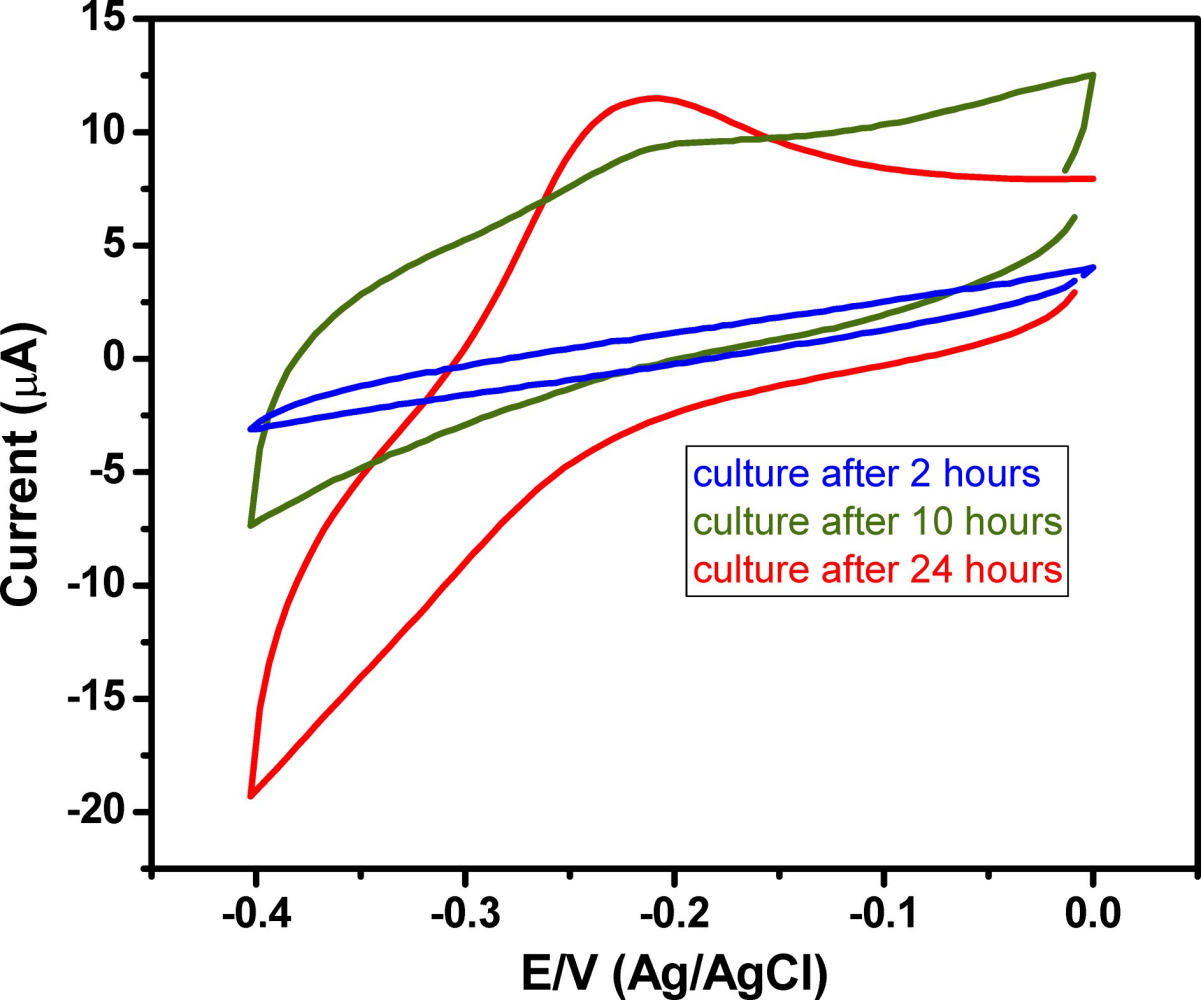
Electrochemical detection of pyocyanin in *P*. *aeruginosa* culture at 37°C after 2, 10 and 24 hours of incubation.

In this study, different samples from the *P*. *aeruginosa* cultures were gathered during log and stationary phases. The pyocyanin was released from *P*. *aeruginosa* culture and detected electrochemically by PANI/Au NPs modified ITO after 2, 10, and 24 hours. The results in **Fig 7** showed that pyocyanin was not released in the culture after 2 hours of incubation (OD at 600 nm was 0.1, so the culture was in the log phase). Pyocyanin could be detected after 10 hours as the culture reached the stationary phase. This result is consistent with that obtained by Cabeen 2014 who reported that the release of pyocyanin is controlled by the quorum detection system that doesn't exist in the early stage of the culture [53].

### 3.6. Electrochemical testing of bacterial cultures

**Fig 8:**
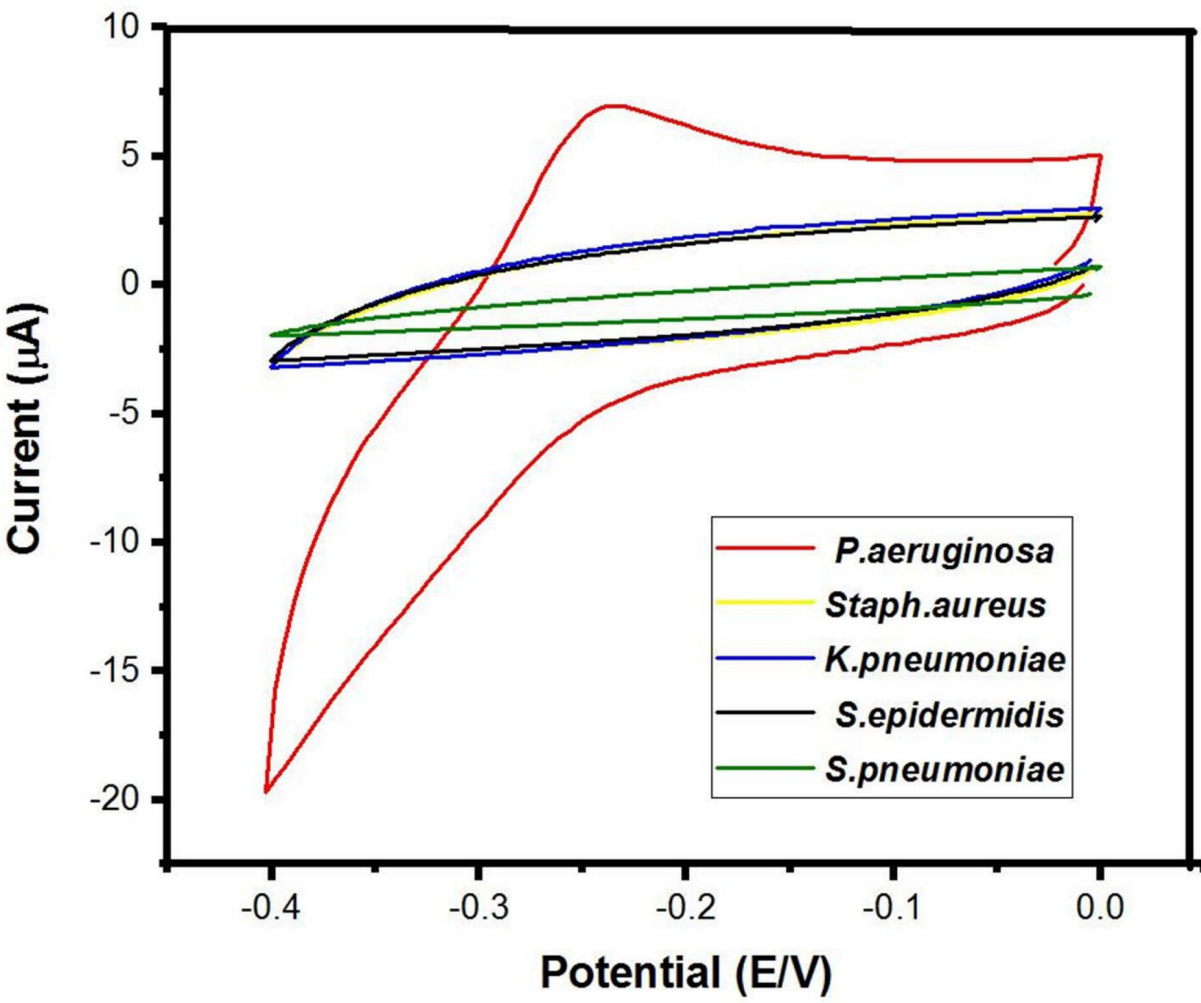
Cyclic voltammetry of different bacterial cultures after one day of growth at 37 ^ₒ^C.

In this application, the major concern was the possible interference of other bacteria producing redox-active molecules with the response of the sensor in the potential window of pyocyanin. Therefore, a variety of clinically-relevant bacteria (**Fig 8**) were electrochemically measured by the PANI/Au NPs modified ITO after 24 hours of growth. The above results indicated that this sensor demonstrated high selectivity to pyocyanin. A remarkable oxidation peak at -0.23 V was originated from the *P*. *aeruginosa* strain, and no additional redox-active peaks were detected for the other tested bacteria within this potential window. The possibility of a false positive identification of *P*. *aeruginosa* using this method is doubtful because the other pathogens didn't show a detectable peak in the potential window of pyocyanin. This endorses that only *P*. *aeruginosa* was generating redox-active particles between the tested bacteria.

### 3.7. Electrochemical sensing of P. aeruginosa strain in clinical samples

**Fig 9:**
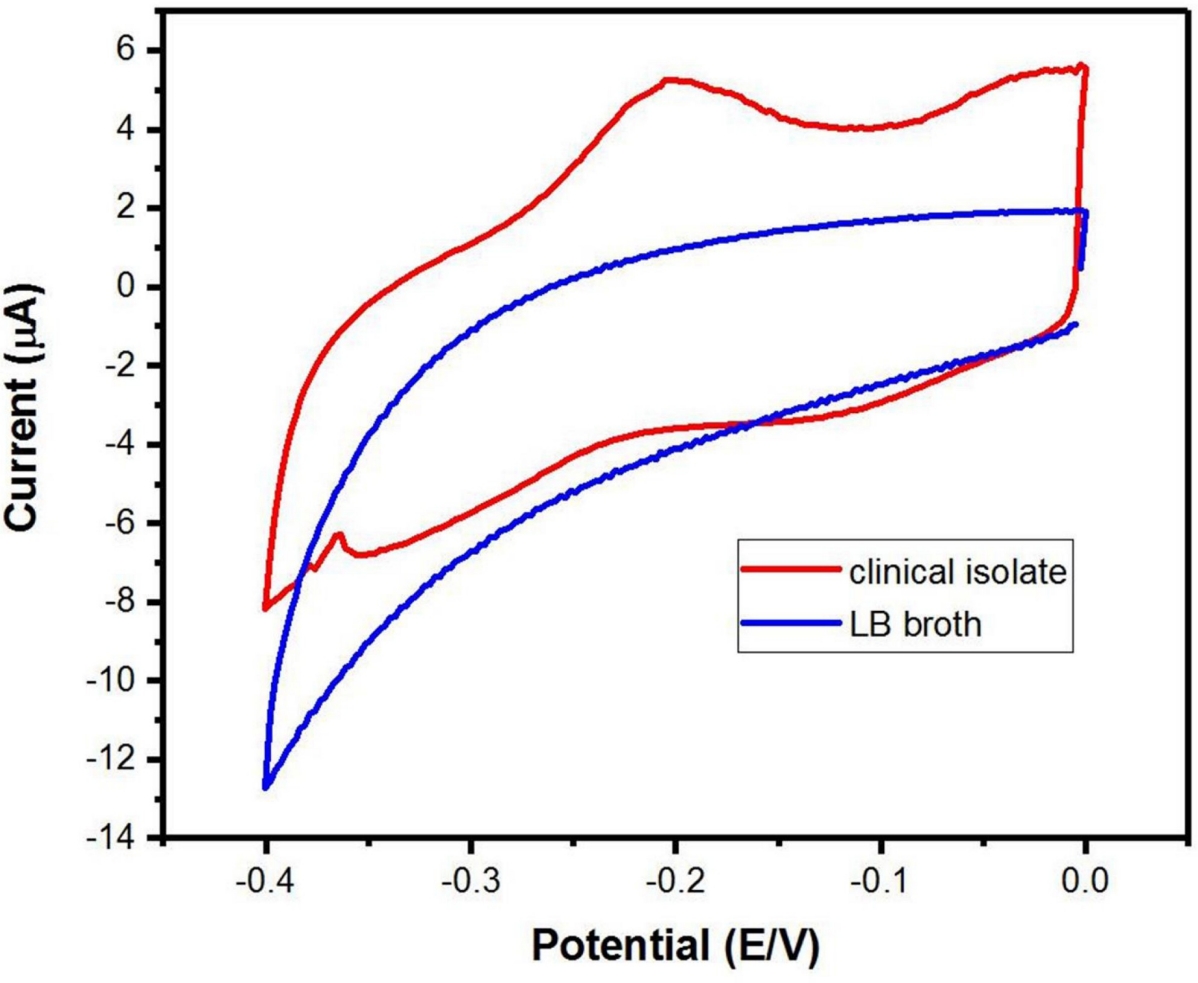
Cyclic voltammetry of a clinical isolate of *P*. *aeruginosa*.

Clinical *P*. *aeruginosa* isolates were cultured and incubated at 37°C for 24 hours; then the direct electrochemical sensing was performed. All *P*. *aeruginosa* isolates were having a positive test result of electrochemical detection by PANI/Au NPs modified ITO electrode. The observation of an electrochemical peak at -0.20 V was shown in **Fig 9**, which is a characteristic oxidation peak of the pyocyanin indicating that the examined sample was containing *P*. *aeruginosa*. The negative control was uninoculated LB broth, which didn't show the pyocyanin redox-active oxidation peak.

## 4. Conclusions

In summary, PANI/Au NPs decorated ITO electrode was fabricated by the electrodeposition of Au NPs onto the surface of the ITO substrate based on CV technique, followed by covering the surface with a layer of PANI. The prepared electrode showed 100% sensitivity, selectivity, and a low detection limit for pyocyanin. The capability of the PANI/Au NPs modified ITO sensor to detect pyocyanin released in *P*. *aeruginosa* culture will aid in the fast, precise detection of pyocyanin biomarker and diagnosis of *P*. *aeruginosa* infections especially in critically ill patients. Consequently, this will achieve a rapid appropriate treatment and reduce the emergence of resistance made by the empirical treatment.

## Supporting information

S1 File Analysis of the particles sizes by using ImageJ (IJ 152) analysis. (XLS)
